# *Ich sehe was, was du auch siehst. *Über die Möglichkeiten von Augmented und Virtual Reality für die digitale Beteiligung von Bürger:innen in der Bau- und Stadtplanung

**DOI:** 10.1365/s40702-021-00772-6

**Published:** 2021-08-26

**Authors:** Jonas Fegert, Jella Pfeiffer, Pauline Reitzer, Tobias Götz, Anuja Hariharan, Nadine Pfeiffer-Leßmann, Patrick Renner, Thies Pfeiffer, Christof Weinhardt

**Affiliations:** 1 Information Management & Analytics, FZI Forschungszentrum Informatik, Berlin, Deutschland; 2 Professur für Digitalisierung, E-Business und Operations Management, Justus-Liebig-Universität Gießen, Gießen, Deutschland; 3 Lehrstuhl für Digitalisierung, E-Business und Operations Management, Justus-Liebig-Universität Gießen, Gießen, Deutschland; 4 CAS Software AG, Karlsruhe, Deutschland; 5 Neuland Medien GmbH & Co KG, Rheda-Wiedenbrück, Deutschland; 6 Raumtänzer GmbH, Rheda-Wiedenbrück, Deutschland; 7 Institute of Information Systems and Marketing, Karlsruher Institut für Technologie, Karlsruhe, Deutschland

**Keywords:** Bürgerbeteiligung, E‑Partizipation, Augmented Reality, Virtual Reality, Bauplanung, Stadtplanung, Public Participation, E‑Participation, Augmented Reality, Virtual Reality, Construction Planning, Urban Planning

## Abstract

Digital Government eröffnet Möglichkeiten, Verwaltungs- und Regierungsprozesse kritisch zu reflektieren und sie entsprechend neu zu denken. Oblagen Bürgerbeteiligungsprozesse in der Vergangenheit zahlreichen Hürden, bietet die e‑Partizipation Möglichkeiten, sie mit modernen Technologien zu verbinden, die eine niedrigschwellige Teilhabe ermöglichen. In dem Forschungsprojekt Take Part, gefördert durch das Bundesministerium für Bildung und Forschung, werden innovative Formen der Beteiligung von Bürger:innen in der Stadt- und Bauplanung mithilfe von Augmented und Virtual Reality (AR und VR) erforscht. Dabei geht es vor allem darum, neue Anreize zu schaffen, Bürger:innen zur Beteiligung zu motivieren und durch diese das Konfliktpotential um Bauprojekte zu reduzieren. Mithilfe der innerhalb von Take Part entwickelten App können Bürger:innen Bauvorhaben diskutieren, Feedback geben oder über sie abstimmen, während sie dabei den Beteiligungsgegenstand anschaulich in AR und VR präsentiert bekommen. Zugleich können auch Initiator:innen mithilfe eines Partizipationsökosystems die Beteiligung im jeweiligen Bauvorhaben konfigurieren, indem sie sowohl vorhandene Module kombinieren und konfigurieren, als auch passende Dienstleistungen, wie beispielsweise 3D-Modellierungen, einkaufen. In diesem Beitrag sollen die konkreten technologischen Entwicklungen (u. a. Outdoor-AR-Tracking und räumlich verankerte Diskussionen), sowie das Partizipationsökosystem (Dienstentwicklungs- und Ausführungsplattform) vorgestellt werden. Erstmalig soll so der entwickelte Prototyp umfassend dargestellt werden. Auf die Herausforderung, eine e‑Partizipations-App zu entwickeln, die die Möglichkeit bietet, verschiedene Interaktionskonzepte ineinander zu integrieren und gleichzeitig eine überzeugende User-Experience bietet, soll ebenfalls eingegangen werden. Anschließend wird das Potenzial einer solchen Lösung für die digitale Mitbestimmung in lokaler Verwaltung vor allem in Bezug auf gesteigerte Vorstellungskraft und Motivation zur Teilhabe für Nutzer:innen diskutiert und in den Kontext der Covid-19 Pandemie gesetzt.

## Abstract (en)

Digital government opens up opportunities to critically reflect on administrative and government processes and to rethink them accordingly. In the past, citizen participation processes were subject to numerous challenges, but e‑participation offers opportunities to combine them with modern technologies that enable low-threshold participation. In the research project Take Part, funded by the German Federal Ministry of Education and Research, innovative forms of citizen participation in urban and construction planning are being researched using augmented and virtual reality (AR and VR). The main goal is to create new incentives to motivate citizens to participate and to reduce the possible conflict in construction projects. With the help of the application developed within Take Part, citizens can discuss construction projects, give feedback or vote on them, and at the same time have the object of participation vividly presented in AR and VR. Furthermore, initiators can configure the participation ecosystem to find combinable applications for participation in the respective construction project and purchase suitable services, such as 3D modeling. This paper will present the concrete technological developments (incl. outdoor AR tracking and spatially anchored discussions), as well as the participation ecosystem (service development and implementation platform). For the first time, the developed prototype will be presented in detail. The challenge of developing an e‑participation app that offers the chance to integrate the interaction concepts of different technologies and at the same time provides a compelling user experience will also be described. Finally, the potential of such a solution for digital participation in local government will be discussed, especially in regard of the increased power of imagination and motivation to participate for the user and additionally put into the context of the Covid-19 pandemic.

## Einleitung

In Zeiten der Urbanisierung, in der Menschen weltweit vermehrt in Städte ziehen (United Nations et al. 2019), steht die Nutzung öffentlichen Raums im großen Maße zur Debatte. Werden bei Bauprojekten nicht alle beteiligten Stakeholder miteinbezogen, so können Konflikte entstehen, die eine gesellschaftliche Spaltung vorantreiben können. So ist beispielsweise die Belastung der öffentlichen Hand aufgrund steigender Baukosten ein ernstzunehmendes Problem. Die Konsequenzen exklusiver Bauplanung zeigten sich in den vergangenen Jahren weltweit an diversen Konflikten. 2019 kam es in New York City zu der Debatte um eine neue Amazon Firmenzentrale, bei der die Proteste gegen die damit einhergehende Gentrifizierung des Stadtteils zu einem Abbruch des Bauprojekts führten. In Deutschland war vor allem der Konflikt um die Neugestaltung des Stuttgarter Hauptbahnhofs ausschlaggebend für eine eingehendere Beschäftigung von Bürger:innenbeteiligung und der Frage nach zeitgemäßen Visualisierungen von Bauvorhaben. Stuttgart 21 kann als eines „der umstrittensten Infrastrukturprojekte in Deutschland“ (Brettschneider 2013) beschrieben werden. Die Neugestaltung des Kopfbahnhofs begann 2010 vor allem durch einen Teilabriss des Gebäudes, der zu zahlreichen Protesten führte, der wiederum eine überregionale Öffentlichkeit zusah. Der Konflikt konnte nur durch ein Schlichtungsverfahren und einen Volksentscheid gelöst werden und in der Konsequenz untersuchten Forscher:innen vermehrt, was in Stuttgart genau schief gelaufen war und wie künftig solche Fehler vermieden werden könnten (Schuster 2013). Ein Grund, der wiederholt in der Forschung auftaucht, ist die schlechte Kommunikation (Baupläne waren bspw. nicht an Ort und Stelle für die Bürger:innen einsehbar) der Initiator:innen, die den Bau als alternativlos darstellten (Thaa 2013). Die mangelnde Einbindung von Bürger:innen, sowie eine intransparente Kommunikation der Initiator:innen kann so Vertrauen in Politik und Verwaltung nachhaltig beeinträchtigen. Dieser lokale Konflikt war für die Entwicklung des im Folgenden dargestellten Forschungsprojektes von Bedeutung. Das vom Bundesministerium für Bildung und Forschung geförderte Projekt Take Part folgt der Idee mithilfe zeitgemäßer Visualisierung von Bauvorhaben, die Interaktion zwischen Initiator:innen und Bürger:innen zu verbessern. Mithilfe von Augmented und Virtual Reality (AR und VR) sollen Bürger:innen ermutigt werden, sich an Bau- und Stadtplanung zu beteiligen. AR- und VR-Technologien können durch entsprechende Hardware Räume immersiv erlebbar machen. Im Ergebnis soll so die App Konfliktpotenziale von Bauprojekten frühzeitig erkennen und verhindern. Die Forschungsfrage, die das Projekt begleitet lautet daher:

*Wie können Bürger:innen mithilfe von AR- und VR-Technologien frühzeitig und niedrigschwellig über Bauvorhaben informiert werden und kann dadurch ein Anreiz zur Bürgerbeteiligung geschaffen werden, um zu Entscheidungen beizutragen, die spätere Konflikte vermeiden*?

Mit dem Ziel dieser Forschungsfrage nachzugehen, fand sich 2018 ein interdisziplinäres Konsortium, bestehend aus Forschungseinrichtungen (FZI Forschungszentrum Informatik, Justus-Liebig-Universität Gießen, Karlsruher Institut für Technologie) und Unternehmen (Raumtänzer GmbH, Neuland Medien GmbH & Co KG und CAS Software AG) zusammen. Dieses arbeitete zwischen 2018 und 2021 an der Entwicklung und Erforschung der App Take Part, die in diesem Artikel als Prototyp erstmals umfassend vorgestellt werden soll. Im ersten Teil des Artikels werden die theoretischen Grundlagen bezüglich E‑Partizipation und AR und VR beleuchtet und somit ein Ausgangspunkt für die Beantwortung der Forschungsfrage geschaffen. Das darauffolgende Kapitel führt die beiden Themengebiete zusammen und erarbeitet, welche Chancen AR- und VR-Technologien in Bezug auf Bürger:innenbeteiligung eröffnen. Anschließend werden die Take Part-App (finaler Prototyp) und das Partizipationsökosystem vorgestellt und es wird beschrieben, wie damit den geschilderten Herausforderungen begegnet wird.

## Theoretische Grundlagen

### E-Partizipation

E‑Demokratie beschreibt das weitgefasste Konzept der Nutzung von Informations- und Kommunikationstechnik, um demokratische Beteiligung und Prozesse zu fördern und zu stärken (Macintosh 2004). E‑Partizipation kann laut Macintosh (2004) als der Teil der e‑Demokratie verstanden werden, durch die die Bürger:innen direkt in die demokratische Entscheidungsfindung eingebunden werden. Sie ist keine Technologie und es geht bei ihr nicht primär um die Nutzung von Technologien innerhalb der politischen Wahlprozesse (e-Voting), sondern vielmehr um eine Form der Bürger:innenbeteiligung die mit ihrem kollaborativen Charakter über das alljährliche Wählen hinausgeht (Sanford und Rose 2007).

Die International Association for Public Participation liefert ein umfassendes Modell zur Kategorisierung von Bürger:innenbeteiligung mithilfe verschiedener Ebenen (International Association for Public Participation 2018). In ihrem Partizipationsspektrum hat jede Ebene der Beteiligung („inform“, „consult“, „involve“, „collaborate“ und „empower“) unterschiedliche Auswirkungen auf die Entscheidungsfindung. Dieses für die klassische Bürger:innebeteiligung angelegte Modell lässt sich auch auf die e‑Partizipation übertragen (Nabatchi 2012; Nelimarkka et al. 2014; Wirtz et al. 2018). Die generelle positive Wirkung von Beteiligungsstrukturen auf beispielsweise die Motivation, Leistung und Zufriedenheit von Mitarbeitenden (Wegge et al. 2010) gilt es zu nutzen und auf die Stadtplanung zu überführen. Wolf et al. (2020) gehen auf eben diese Forderung ein und stellen bereits entwickelte Ansätze von Elementen der e‑Partizipation für die Stadt- und Bauplanung vor. Als Argumentationsgrundlage für die Notwendigkeit einer intensivierten Forschung in dem Bereich, wird das Beteiligungsparadoxon aufgeführt, welches den Konflikt zwischen einem gesteigerten Interesse an Beteiligung und der zeitgleichen Abnahme von Optionen der Beteiligung für Betroffene im Laufe eines Planungsprozesses beschreibt. Im Kontext der Stadt- und Bauplanung schlägt sich dieser Konflikt darin nieder, dass zu einem frühen Planungszeitpunkt die Beteiligungsbereitschaft der Betroffenen meist niedrig ist, dem liegt aber nicht ein fehlendes Interesse an den Bauprojekten, sondern vielmehr das überfordernde Abstraktionslevel zu Grunde (Wolf et al. 2020). Um dem Beteiligungsparadoxon und seinen Konsequenzen entgegenzuwirken, verweisen die Autoren auf die Chance von e‑Partizipation und plädieren dafür die theoretischen Überlegungen mit evidenzbasierten Methoden zu prüfen.

### Augmented und Virtual Reality

In der praktischen Anwendung von AR wird die unmittelbare physische Umgebung mit virtuellen Elementen ergänzt (Azuma 1997). Zudem ermöglicht AR eine Erweiterung der wahrnehmbaren Realität um weitere Informationen wie Textobjekte oder Bilder (Kind et al. 2019). VR lässt hingegen die User:innen in eine interaktive 3D-Umgebung eintauchen (Immersion) und platziert sie:ihn in eine virtuelle Welt (Wexelblat 1995; Suh und Lee 2005). Oftmals wird dies mit Hilfe von Head-Mounted-Displays (HMDs) erreicht, die mithilfe einer Anzeige die virtuelle Umgebung vor das Blickfeld der anwendenden Person setzen (Meißner et al. 2020; Fegert et al. 2020). Suh und Lee (2005) zeigten auf, dass ein VR-Interface Interesse und Wissen zu einem Produkt fördern kann und Peukert et al. (2019) zeigten, dass Immersion zu mehr Spaß bei der Benutzung einer Anwendung führen kann. Auch in der Bau- und Immobilienbranche wird VR eingesetzt, um Interesse an Bauprojekten zu erzeugen (Whyte 2003; Barnes 2016). Wolf et al. (2020) formulieren die Chancen des Einsatzes von AR- und VR-Anwendungen vor allem in den frühen Stadien der Stadt- und Bauplanung. Nach Wolf et al. (2020) könnten AR und VR Technologien als eine Ergänzung von Beteiligungsformaten eingesetzt werden um Verständlichkeit, Nachvollziehbarkeit, Kollaboration und Interaktion von Beteiligten in Planungsprozessen zu fördern. Sie formulieren die Notwendigkeit zukünftiger Forschung bezüglich AR- und VR-Nutzung für konkrete Anwendungsszenarien und den Anforderungen verschiedener Beteiligungsebenen, wozu dieser Artikel und das Projekt Take Part einen ersten Beitrag leistet.

## Berührungspunkte und Herausforderungen

E‑Partizipation bietet Möglichkeiten, Beteiligungsprozesse zu überdenken und technologisch neue Möglichkeiten der Partizipation zu eröffnen. Interessant ist hierbei, welche Funktion konkrete technologische Entwicklungen (u. a. Outdoor-AR-Tracking und räumlich verankerte Diskussionen), sowie das Partizipationsökosystem (Dienstentwicklungs- und Ausführungsplattform) in Prozessen der e‑Partizipation einnehmen können, um diese niedrigschwelliger, interessanter und motivierender zu gestalten. Ein Ansatz von Nuernberger et al. (2016) kann beispielsweise aufgegriffen werden, nämlich wie mithilfe von Annotationen kleine Zeichnungen auf mobilen Geräten präzise in 3D auf einem Gebäude platziert werden und zugleich Zeichnungen, Textfelder, Bilder und Audiokommentare innerhalb der Visualisierung hinzugefügt werden können.

Um ein IT-Artefakt zu entwickeln, welches nah an den Bedürfnissen der Anwender:innen gestaltet wird, wurde anfangs auf einen Design-Science-Ansatz von Pfeffers Peffers et al. (2007) zurückgegriffen. Um der steigenden Komplexität gerecht zu werden, wurde dieser während des Forschungsprozesses durch den Ansatz von Kuechler und Vaishnavi (2008) ersetzt. Die Drei-Zyklus-Ansicht für Design-Science-Forschung ermöglicht die Entwicklung des Prototypen transparent und strukturiert zu halten. Um das Problem zu definieren wurde eine qualitative Studie mit beteiligten Stakeholder:innen durchgeführt und später das Artefakt evaluiert (Fegert et al. 2020). Somit folgte die Entwicklung von Take Part einem Ansatz, der die Nutzer:innen ins Zentrum stellte.

**Aus Sicht der Bürger:innnen** können Technologien wie AR und VR nicht nur eine inklusive und niedrigschwellige Teilhabe fördern, sondern auch die Motivation zur Beteiligung erhöhen und das Vorstellungsvermögen unterstützen (Fegert et al. 2020). Bürger:innenbeteiligung erfordert, dass alle Beteiligten eine ähnliche Vorstellung von den betreffenden Konzepten haben. Gerade in puncto Wissensstand und Expertise ist es jedoch vorhersehbar, dass Wissen der Stakeholder:innen ungleich verteilt ist und die individuelle Interpretation von präsentierten Konzepten schnell auseinander gehen kann (Rockmann et al. 2015). Wenn dieser Prozess nicht durch klare Kommunikation abgefangen wird, sind Missverständnisse kaum zu vermeiden, welche wiederum einer erfolgreichen Teilhabe im Weg stehen. Studien belegen: Technologien wie AR und VR können diesen Prozess unterstützen, indem sie den Interpretationsspielraum der Beteiligten eingrenzen (Goudarznia et al. 2017). Macintosh nannte bereits 2008 Visualisierungen, darunter 3D-Umgebungen und VR, als eine wichtige Forschungsaktivität der e‑Partizipation. Die Idee die Technologien zu nutzen, begann mit der Verwendung der Technologien als partizipatives Element in der Stadt- und Bauplanung (Allen et al. 2011; Rockmann et al. 2015; Goudarznia et al. 2017; Wolf et al. 2020). Auf diese Forschung baut Take Part auf.

Anders als die genannte Forschung (und bereits etablierte e‑Partizipationstools) versucht Take Part Beteiligung mithilfe von AR und VR auf allen Ebenen des Partizipationsspektrums („information“, „consultation“, „involvement“, „collaboration“ und „empowerment“) zu ermöglichen. Um dieses komplexe Unterfangen für die Bürger:innen überschaubar zu gestalten, ist ein Design, dass sich durch Benutzer:innenfreundlichkeit auszeichnet, unabdingbar.

Während das immersive Partizipationsumfeld auf die Nutzung durch die Bürger:innen ausgerichtet ist, soll dabei nicht übersehen werden, dass die **Mitwirkung der Initiator:innen** ebenfalls möglichst umfassend angeboten werden soll. Bauprojekte haben die Eigenschaft, erst dann Aufsehen zu erregen, wenn sie sich bereits in der Umsetzungsphase befinden. In diesem Stadium ist es oft schwer bis unmöglich nachträglich die Bedürfnisse und Anregungen betroffener Bürger:innen miteinzubeziehen und dadurch wird die Chance verpasst Anregungen der Bevölkerung (*Wisdom of the Crowd*) zu bedienen. Kreativer Input und Expert:innenwissen bleibt also in diesem Kontext ungenutzt. AR und VR können dieser Problematik entgegenwirken, indem sie den Bauprozess virtuell in andere Zeitstadien verschieben. Damit kann das sonst zuspätkommende Feedback rechtzeitig eingefangen und Co-Kreation ermöglicht werden (Jutraž und Moine 2016; Imottesjo und Kain 2018). Ebenfalls relevant für Initiator:innen ist, dass Computer-Aided-Design (CAD) bereits von Architekt:innen in der Bauplanung genutzt wird. Daher ist eine technische Grundlage vorhanden, die eine Anpassung für die Nutzung in AR und VR im Kontext von digitaler Beteiligung relativ einfach gestaltet (Lorenz et al. 2016).

Wie bereits das Partizipationsspektrum vermuten lässt, sind die Möglichkeiten Bürger:innen durch (digitale) Beteiligung in die Bauplanung einzubinden vielfältig. Je nach Art des Bauprojektes, seiner geographischen Lage und gesellschaftlichen Stellung, und auch den Interessen der Innitiator:innen unterscheiden sich die Beteiligungsformate in ihren Anforderungen. Die Konfiguration von Systemen der e‑Partizipation ist daher wissensintensiv. Sie zu ermöglichen, erfordert aus diesem Grund auch spezifische technische, organisatorische und wirtschaftliche Expertise, die in Abhängigkeit zu dem gewählten Format steht. Initiator:innen stehen somit nicht nur vor der Herausforderung das passende Beteiligungsformat zu dem entsprechenden Projekt zuordnen zu müssen, sondern auch vor dem Problem einer fehlenden Expert:inneninfrastrukur für die technische Umsetzung. Hier soll die Idee eines Partizipationsökosystems Abhilfe leisten.

**Abb. 1:**
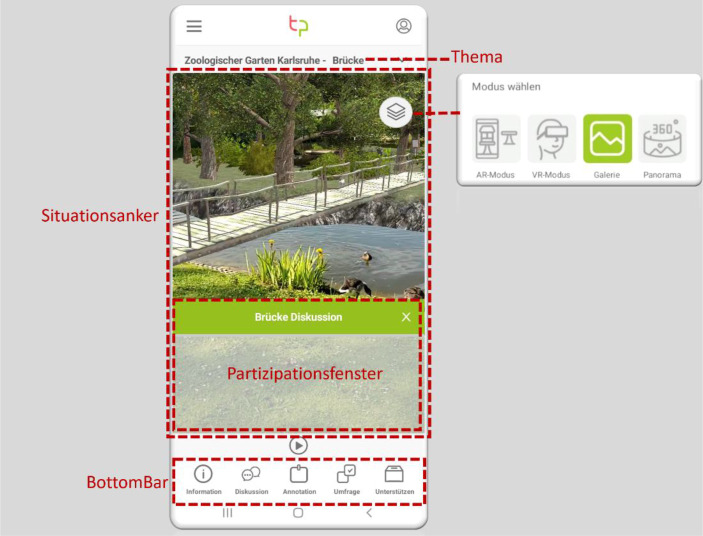
Der strukturelle Aufbau der Take Part-App

Als Use Case dient im Projekt Take Part der Zoologische Stadtgarten Karlsruhe. Er ist eine Einrichtung der Stadt Karlsruhe, plant jedoch mit Spenden seines Fördervereins, eine Insel in dem parkartigen Zoogelände für die Nutzung als freies Gehege für Kattas, eine Lemuren-Art, umzubauen. Auf die Entwürfe beziehen sich auch die Abb. 1, 2 und 3.

**Abb. 2:**
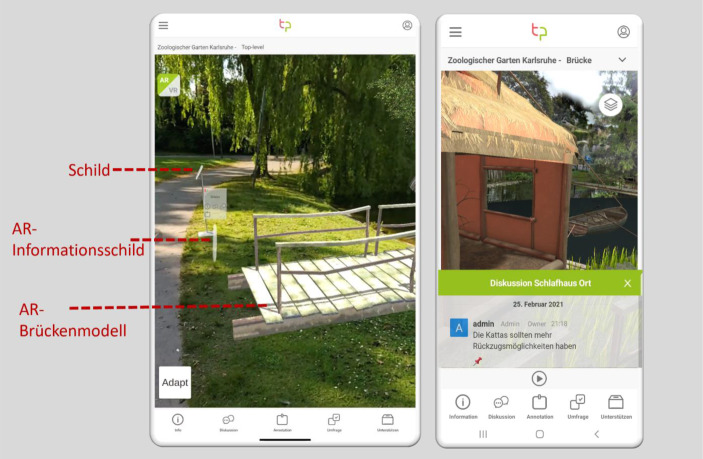
Die Take Part-App mit seinen AR (**a**) und VR (**b**) Sichtweisen

**Abb. 3:**
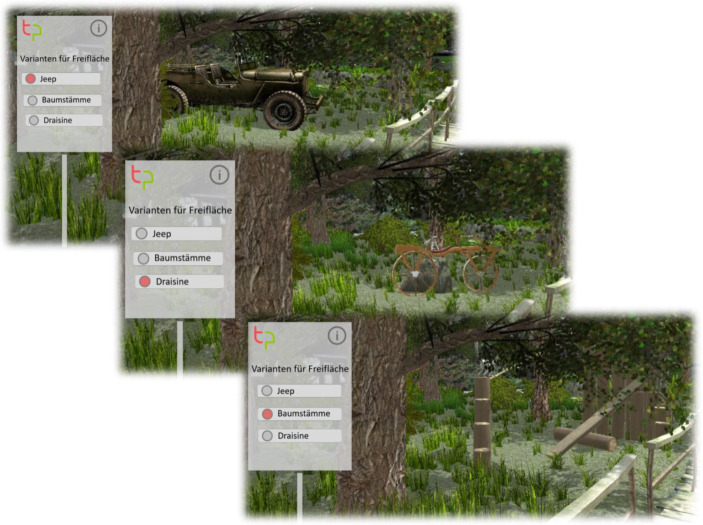
Eine Abstimmung zu verschiedenen Versionen einer Außengestaltung

## Lösungsansätze und ihre Wirksamkeit

Innerhalb des Take Part-Projektes wurde ein Prototyp entwickelt, welcher die oben beschrieben Chancen von AR und VR in der Bürger:innenbeteiligung versucht bestmöglich aufzugreifen. Dieser soll präsentiert werden und gleichzeitig soll auf damit verbundene Herausforderungen eingegangen werden. Die Take Part-App dient dazu, die Initiator:innen und Bürger:innen zusammen zu bringen. Sie bietet eine gemeinsame Plattform zum Austauschen und zum Aufbau eines gemeinsamen Kontexts. Die initiierende Person oder Institution konfiguriert das Projekt und damit die Module in der App so, dass es ihren Wünschen in Bezug auf die Partizipationsmöglichkeiten entspricht. Der Aufbau der Take Part-App spiegelt diesen Ansatz wider und passt sich dynamisch an. Im folgenden Kapitel werden die konkreten technologischen Entwicklungen beschrieben und es wird erarbeitet wie diese den oben genannten Anforderungen gerecht werden.

Initiator:innen gestalten den oberen Bereich der App, indem sie beispielsweise Inhalte des Haupthintergrundfensters als Situationsanker festlegen (Abb. 1). Hier können, um eine gemeinsame Wissensbasis zu schaffen, Bilder hochgeladen, Panorama-Touren verlinkt und AR- oder VR-Modelle eingestellt werden. Die Bürger:innen können in die geplanten Modelle eintauchen und mittels Diskussionen, Annotationen und Umfragen partizipieren. Zudem können die Initiator:innen durch das Vorgeben von Themen dem Projekt eine Struktur verleihen.

Die *BottomBar* der App spannt die verschiedenen Ebenen der Partizipation (s. Abschnitt 2.1) der Bürger:innen auf (Abb. 1). Als ein Ansatz eines nutzer:innenzentrierten Designs wurden zudem Situationsanker entwickelt, die Partizipationsmodule wie Information, Diskussion, Annotation, Umfrage und Unterstützen in der immersiven Umgebung verankern. Das angewählte Partizipationsmodul legt sich als transparentes Fenster (siehe Partizipationsfenster in Abb. 1) über den Hintergrundkontext, sodass die Bürger:innen im gewünschten Situationsanker interagieren können. Die Grundsatz-Designanforderungen der Take Part-App folgen dabei den Maximen: Orientierung schaffen, Situiertheit, Vertrautheit, Multiperspektive (alle Informationen und Konzepte direkt im parallelen, einfachen Zugriff), Aktualität (Information über Neuigkeiten seit letztem Aufruf), Rechteverwaltung (zielgruppen-spezifische Zugriffe) und klare Trennung zwischen Informationen von Initiatoren und Bürger:innen. Durch die Umsetzung dieser Anforderungen wird vor allem die Nutzer:innenfreundlichkeit für Bürger:innen sowie Initiator:innen sichergestellt.

### Umsetzung der immersiven Beteiligung

Eine besondere Herausforderung bei baulichen Planungsvorhaben besteht in dem Übergang von 2D-Plänen zu einer 3D-Einordnung von Situationen aus einer personenbezogenen Perspektive. Um dies zu ermöglichen, bietet die Take Part-App AR- und VR-Visualisierungen. Stehen Bürger:innen vor einer geplanten Baufläche, so können sie sich durch Scannen eines Markers, beispielsweise eines Schilds, mittels AR direkt das geplante Objekt in der Realwelt anzeigen lassen. Somit kann die Person genau die Maße und das Einpassen des Bauobjekts in die Umgebung abschätzen. Abb. 2 (links) zeigt das reale Schild zum Scannen, ein virtuelles Informationsschild und ein virtuelles Modell der geplanten Brücke. Für nicht zugängliche Bereiche auf der Insel können User:innen in das VR-Modell wechseln und sich damit direkt auf die Insel in das Modell *teleportieren*. In der VR kann der/die Bürger:in sich bewegen und überall umschauen. Informationsschilder bieten themenbezogene Informationen und dienen als Partizipationsorte (Abb. 2 links). Die App liefert dem:der Nutzer:in ein Partizipationserlebnis, welches Informationen nicht nur realistisch vorstellbar macht, sondern durch Interaktivität auch zu Kreativität und Beteiligung motivieren kann.

Oft besteht bei Diskussionen das Problem, dass die Beteiligten nicht den gleichen Wissensstand beziehungsweise die gleichen Vorstellungen haben. Diskussionen in einem Forum finden zudem zumeist asynchron statt, was diese Problematik noch verschärft. Daher bietet die Take Part-App das Konzept der Situationsanker, um Beiträge in einem Kontext zu verankern. Wenn sich Bürger:innen beispielsweise ein neues Modell eines Katta-Schlafhauses ansehen und äußern möchten, dass die Kattas zu wenig Rückzugsmöglichkeiten hätten, so können sie den Kommentaren eine Verankerung mitgeben, an welcher Stelle des Geheges ihnen dieser Gedanke kam. Andere Bürger:innen können den Link, in Abb. 2 (rechts) repräsentiert durch den Pin, abrufen und werden so direkt in den Kontext der Äußerung versetzt. Durch die dezente Verlinkung des Situationsankers wird der Diskussionsfluss nicht gestört, die Nachvollziehbarkeit von Äußerungen jedoch erhöht. Die Take Part-App integriert *Rocket.Chat* als Tool für Diskussionen, ergänzt dieses jedoch durch JavaScript-Erweiterungen, um diese spezifischen Zusatzfunktionalitäten zu ermöglichen.

Möchten Bürger:innen unabhängig von einer Diskussion eine Anmerkung machen, so können sie Annotationen (z. B. Audiokommentare sowie ergänzende Bilder oder Kommentare) direkt an einem Ort in der AR-/VR-Welt verankern. Eine weitere Funktionalität, die die Take Part-App unterstützt, ist die Varianten-Visualisierung. Die Initiator:innen können Konfigurator-Schilder aufstellen, die für Bürger:innen die Bauvorhaben direkt visuell erfahrbar machen, ohne einen komplizierten Transfer aus technischen Zeichnungen leisten zu müssen. Basierend auf der Variantendarstellung kann der Initiator auch zu einer Umfrage überleiten, um ein Meinungsbild einzuholen oder eine Abstimmung vorzunehmen. Die Bürger:innen können die Varianten kommentieren oder up-down voten, sowie auch eigene Vorschläge mit konkreten Bildbeispielen anheften und so auch Alternativen vorschlagen. Die Situationsanker konkretisieren somit die Beiträge der Bürger:innen indem sie sie den infrage stehenden Objekten (z. B. dem Schlafhaus der Kattas) zuordnen können. Somit liefern sie eine Wissensgrundlage und wirken dem Wissensvorsprung zwischen Initiator:innen und Bürger:innen entgegen. Missverständnisse über Baupläne können durch diese Funktion vorgebeugt werden.

Aufgrund der unterschiedlichen Technologien besteht eine Herausforderung bei der Umsetzung von AR- und VR-Inhalten in mobilen Apps darin, gleichzeitig eine optimale User Experience bei der App-Nutzung sowie eine optimale Performance und Qualität der immersiven Darstellungen sicherzustellen. Die Take Part-App löst dieses Problem, indem es zwei technologische Ansätze kombiniert. Die grundlegende App wurde nativ mit dem *Xamarin*-Framework umgesetzt. So kann eine stimmige User Experience garantiert werden. Die immersiven Inhalte wiederum wurden mit der *Unity*-Spiel-Engine entwickelt. Diese erlaubt flexible und performante Umsetzung von 3D-Anwendungen, auch für AR und VR: Das ARFoundation Framework vereint die nativen AR-Bibliotheken *ARCore* (Android) und *ARKit *(iOS) und stellt ein stabiles Tracking sicher. Die in *Unity* erstellten Inhalte werden als Plugin in der *Xamarin*-basierten App geladen und komplett integriert.

### Umsetzung des Partizipationsökosystems

Die Initiator:innen bekommen in der Take Part-App mithilfe eines sogenannten Partizipationsökosystems, das man sich wie einen digitalen Marktplatz vorstellen kann, die Möglichkeit ein Partizipationsumfeld zu konfigurieren. Das Partizipationsökosystem bringt Technologieanbieter:innen, Branchen-Softwarespezialist:innen und Content-Betreiber:innen zusammen und ermöglicht es Initiator:innen Partnerschaften zu gestalten und notwendige Expertise nach projektspezifischem Bedarf zu integrieren. Durch wiederverwendbare Technologiebausteine können innovative Lösungen für unterschiedliche Zielbranchen entwickelt und eingesetzt werden. Dieses Partizipationsökosystem ermöglicht aus Perspektive der Initiator:innen einen Austausch mit den Bürger:innen zu initialisieren, flexibel zu konfigurieren, zu führen und laufend auszuwerten. Neben dem kreativen Input der Bürger:innen, der sich so für Initiator:innen erschließen lässt, ermöglicht das Partizipationsökosystem eine vereinfachte technische Umsetzung der Beteiligungsformate (z. B. durch die Überführung von CAD-Modellen von Architekten in die AR/VR-Umgebung). Des Weiteren können hier Expert:innen, beispielsweise zur Erstellung von 3D-Modellen, beauftragt und hinzugezogen werden, um bei der Umsetzung des Partizipationsverfahrens zu unterstützen. Durch die Bereitstellung dieser Infrastruktur kann somit auf verschiedenste Anforderungen von Beteiligungsformaten reagiert werden.

**Abb. 4:**
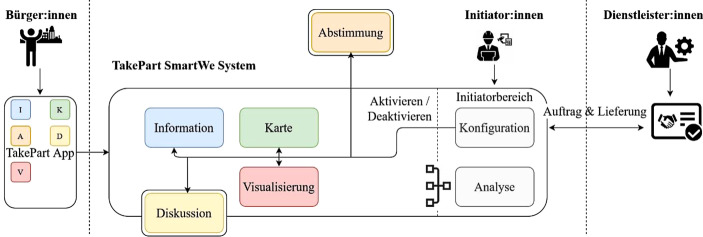
Das Take Part-Partizipationsökosystem

Um die Anforderungen in Bezug auf Anpassbarkeit, Erweiterbarkeit und Flexibilität, die durch die facettenreichen Projekte der Initiator:innen erforderlich sind, umzusetzen, wurde auf ein modulares Systemkonzept gesetzt. Das Take Part-Partizipationsökosystem, basierend auf der *SmartWe* Software der CAS Software AG, stellt einzelne funktionale Komponenten (sogenannte Module) bereit (Abb. 4: Information, Karte, Visualisierung). Zusätzlich lassen sich auch externe Dienste (Abb. 4: Abstimmung unter Verwendung von *LamaPoll*), sowie interne, unabhängige Dienste (Abb. 4: Diskussionsmodul, basierend auf *Rocket.Chat*) als Module einbinden.

**Abb. 5:**
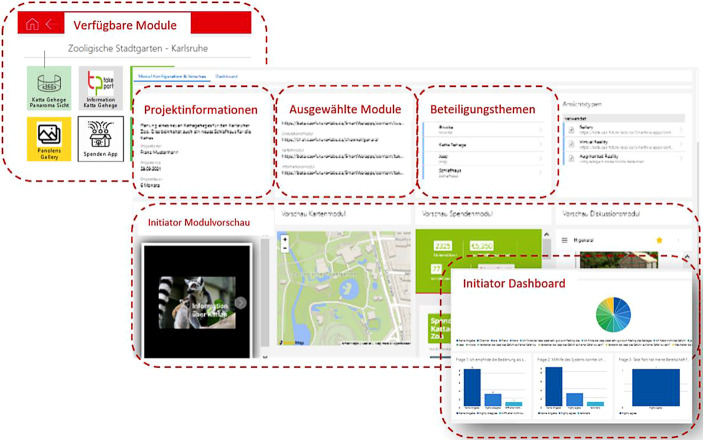
Darstellung Initiator System (Auswahl von Modulen, Projektdatenverwaltung, Modul Vorschau, und Beteiligung Dashboard)

Initiator:innen beginnen die Erstellung ihrer Projekte mit einem Konfigurationsdialog, der ausgehend von den gewünschten Partizipationsebenen eine Modulkombination empfiehlt. Nach der Auswahl der gewünschten Zusammenstellung (Abb. 5) werden die benötigten Kompetenzen für die Inhalte und den Betrieb aller Module vorgestellt. Die Initiator:innen haben ab diesem Zeitpunkt die Möglichkeit benötigte Inhalte oder Verwaltungsaufgaben selbst zu übernehmen oder sie über das Take Part-System an externe Dienstleister zu vergeben. Kommt es zur Vergabe an externe Service Anbieter, so werden Beauftragung, Lieferung und Integration vom Take Part-Partizipationsökosystems übernommen. Die Suche erfolgt basierend auf dem Ort und der hinterlegten Kompetenzen. Über den *SmartWe*-Appstore kann der Initiator die App auch mit vorhandenen CRM-Apps skalieren – beispielsweise mit Projektplanungswerkzeugen oder Besucher:innenberichten. Die gesamten Konfigurationsschritte für die Projektpräsenz können von Initiator:innen jederzeit erneut aufgerufen werden, um strukturelle Änderungen vorzunehmen.

Die Daten aus der Verwendung der Module werden den Initiator:innen in beschränktem Umfang als Datenaggregation in einem Dashboard zur Verfügung gestellt. Damit lässt sich anschließend nicht nur die sinnvolle Konfiguration der Projektpräsenz validieren, sondern zusätzlich können durch Umfrageergebnisse und offene Diskussionsbeiträge auch wichtige Einsichten zum Projekt erzielt werden. Für Initiator:innen stellt die Take Part-App somit einen übersichtlichen, homogenen Einstieg in das Partizipationsökosystem dar. Es besteht zusätzlich keine Notwendigkeit bei einem internen Modul- und Kontextwechsel die Nutzer:innen zu authentifizieren und ermöglicht somit eine schnelle, ungehinderte Interaktion mit den Inhalten.

## Fazit

Ziel des Take Part-Projektes ist die Erforschung von Methoden, um Bürger:innen mithilfe von AR- und VR-Technologien frühzeitig und niedrigschwellig in Beteiligungsformate zu Bauvorhaben einzubinden. Als Lösungsansatz zu der Forschungsfrage stellt dieser Artikel zwei technologische Entwicklungen vor; die Take Part-App und das Partizipationsökosystem. Im Speziellen geht dieser Artikel darauf ein, welche konkreten Anforderungen sich aus einer Nutzung von AR- und VR-Technologien in der Bürger:innenbeteiligung ergeben und wie der entwickelte Prototyp darauf zu reagieren versucht. Eine vorherige Analyse der Möglichkeiten und Herausforderungen ist unbedingt notwendig, um die Entwicklung und das Design des Prototypen danach auszurichten.

Dieser Artikel legt dar, wie die Take Part-App mit AR- und VR-Visualisierungen und Features wie beispielsweise dem Situationsanker eine immersive Beteiligung schafft. In weiteren Studien, die aus dem Forschungsprojekt hervorgingen (Fegert et al. 2020), konnte zudem bestätigt werden, dass die Visualisierung von Bauprojekten mithilfe von AR und VR in unserer App die Nutzer:innen zur Partizipation motiviert und ihre Vorstellungsvermögen unterstützt. Den Visualisierungen gelingt es, eine ähnliche Wissensbasis für die Beteiligten zu schaffen – getreu dem Motto *ich sehe was, was du auch siehst*. Interessanterweise gingen die Take Part Visualisierungen über die bloße Veranschaulichung hinaus, indem sie Nutzer:innen das Gefühl geben konnten virtuell aus der Umgebung der Studie an den Projektstandort versetzt zu werden (Fegert et al. 2020), was als Gefühl der Telepräsenz bezeichnet wird. So können die oftmals trockenen Partizipationsprozesse um Bauvorhaben eine spielerische Dimension erreichen, die niedrigschwellig zu Beteiligung anreizt.

Durch die Integration von hochwertigen AR- und VR-Visualisierungen in ein erprobtes Grundgerüst konnte des Weiteren eine flexible, aber auch verlässliche Basis für das Take Part-Partizipationsökosystem erzielt werden. Die erprobten Konfigurationsmöglichkeiten ermöglichen eine Reihe von Chancen, die bisher Bürger:innen und Initiatoren:innen nicht so unmittelbar zur Verfügung standen. Beteiligungsformate können somit beispielsweise ohne aufwendige technische Expertise an die verschiedenen Anforderungen der Projekte angepasst werden. Diese Konfiguration der Plattform ermöglicht die Erarbeitung eines Bauvorhabens als kollaboratives Produkt und bietet damit bisher nicht vorhandene Zugänglichkeit für Bürger:innen und Initator:innen.

Technisch besteht weiterhin die Notwendigkeit einen Kompromisses zwischen ausreichender Identifizierung der beteiligten Bürger:innen und digitaler Selbstbestimmung zu erreichen. Die Umsetzung der AR- und VR-Funktionen als native, lokale Erweiterung der Take Part-App erwies sich als notwendig, da die erforderlichen Webstandards für diese neuartigen Technologien noch nicht die bestehenden Anforderungen erfüllen.

Die Covid-19 Pandemie hat wie unter einem Brennglas Probleme und Herausforderungen von Gesellschaften aufgezeigt. Die Digitalisierung von öffentlicher Infrastruktur ermöglichte es Staaten schnell auf die Pandemie zu reagieren und gleichzeitig mit ihren Bürger:innen zu interagieren. Hier sehen wir für eine App wie Take Part große Möglichkeiten Partizipation und zwischenmenschliche Einigungsprozesse zu ermöglichen, die komplexes Wissen einfach übersetzen. Mit der innovativen Form der Visualisierung, die wie im Fall von AR lediglich ein Smartphone bedarf, wird eine solide Wissensbasis geschaffen. Daher sehen wir in der vermehrten Nachfrage nach e‑Partizipation in Krisenzeiten (United Nations 2020) eine Chance für unseren Lösungsansatz.

Abschließend muss selbstkritisch beleuchtet werden, dass das Take Part-Partizipationsökosystem zwar für private Bauprojekte geeignet scheint, jedoch der regulatorische Rahmen für öffentliche Bauvorhaben Bürger:innepartizipation erschwert. Das deutsche Baurecht sieht bisher keine digitalen Partizipationsmöglichkeiten vor, gerade wenn es um die konkrete Ausgestaltung von Bauvorhaben geht. Die handelnden Akteur:innen möchten sich zudem oftmals, dies ist auch eine Lehre aus unserem Forschungsprojekt, ihre Kompetenzen nicht streitig machen lassen. Daher konnten wir zwar aufzeigen, dass die digitalen Lösungsmöglichkeiten verfügbar und einsetzbar sind, jedoch wenn es um die konkrete Umsetzung geht, der Wille der Initiator:innen entscheidend ist. Die Etablierung von Technologien wie AR und VR im Massenmarkt wird jedoch auch den Druck auf öffentliche Träger erhöhen, sie innovativ einzusetzen. Denn es liegt nahe, dass Bürger:innen Technologien, die sie im privaten zunehmend nachfragen und schätzen, auch für die Mitgestaltung ihres Umfelds, dem öffentlichen Raum, nutzen wollen. Hierfür haben wir einen Lösungsansatz aufgezeigt.
